# Soybean Oil-Derived Poly-Unsaturated Fatty Acids Enhance Liver Damage in NAFLD Induced by Dietary Cholesterol

**DOI:** 10.3390/nu10091326

**Published:** 2018-09-18

**Authors:** Janin Henkel, Eugenia Alfine, Juliana Saín, Korinna Jöhrens, Daniela Weber, José P. Castro, Jeannette König, Christin Stuhlmann, Madita Vahrenbrink, Wenke Jonas, André Kleinridders, Gerhard P. Püschel

**Affiliations:** 1 Department of Nutritional Biochemistry, Institute of Nutritional Science, University of Potsdam, D-14558 Nuthetal, Germany; jsain@fbcb.unl.edu.ar (J.S.); stuhlman@uni-potsdam.de (C.S.); vahrenbrink@uni-potsdam.de (M.V.); gpuesche@uni-potsdam.de (G.P.P.); 2 German Institute of Human Nutrition, Junior Research Group Central Regulation of Metabolism; D-14558 Nuthetal, Germany; Eugenia.Alfine@dife.de (E.A.); Andre.Kleinridders@dife.de (A.K.); 3 German Center for Diabetes Research (DZD), D-85764 München-Neuherberg, Germany; Wenke.Jonas@dife.de; 4 Department of Biological Sciences, Food Science and Nutrition, Faculty of Biochemistry and Biological Sciences, National University of the Litoral (UNL), Santa Fe S3000, Argentina; 5 Institute of Pathology, Carl Gustav Carus University Hospital Dresden; D-01307 Dresden, Germany; korinna.joehrens@uniklinikum-dresden.de; 6 Department of Molecular Toxicology, German Institute of Human Nutrition; D-14558 Nuthetal, Germany; Daniela.Weber@dife.de (D.W.); Jose.Castro@dife.de (J.P.C.); Jeannette.Koenig@dife.de (J.K.); 7 Department of Medicine, Division of Genetics, Brigham and Women’s Hospital and Harvard Medical School, Boston, MA 02115, USA; 8 Department of Experimental Diabetology, German Institute of Human Nutrition; D-14558 Nuthetal, Germany

**Keywords:** NASH, non-alcoholic fatty liver disease (NAFLD), cholesterol, PUFA, inflammation, oxidative stress

## Abstract

While the impact of dietary cholesterol on the progression of atherosclerosis has probably been overestimated, increasing evidence suggests that dietary cholesterol might favor the transition from blunt steatosis to non-alcoholic steatohepatitis (NASH), especially in combination with high fat diets. It is poorly understood how cholesterol alone or in combination with other dietary lipid components contributes to the development of lipotoxicity. The current study demonstrated that liver damage caused by dietary cholesterol in mice was strongly enhanced by a high fat diet containing soybean oil-derived ω6-poly-unsaturated fatty acids (ω6-PUFA), but not by a lard-based high fat diet containing mainly saturated fatty acids. In contrast to the lard-based diet the soybean oil-based diet augmented cholesterol accumulation in hepatocytes, presumably by impairing cholesterol-eliminating pathways. The soybean oil-based diet enhanced cholesterol-induced mitochondrial damage and amplified the ensuing oxidative stress, probably by peroxidation of poly-unsaturated fatty acids. This resulted in hepatocyte death, recruitment of inflammatory cells, and fibrosis, and caused a transition from steatosis to NASH, doubling the NASH activity score. Thus, the recommendation to reduce cholesterol intake, in particular in diets rich in ω6-PUFA, although not necessary to reduce the risk of atherosclerosis, might be sensible for patients suffering from non-alcoholic fatty liver disease.

## 1. Introduction

The poor reputation of dietary cholesterol traces back to its supposed promoting role in the development of atherosclerosis [1]. However, the impact of dietary cholesterol on atherosclerosis has apparently been largely overestimated [2,3] and more recent recommendations for cardio-protective diets do not include the previously suggested radical reduction of cholesterol intake [4]. Although from the point of view of atherosclerosis, dietary cholesterol might be less relevant than previously assumed, it has reentered the focus of interest because of its potential role in the progression of non-alcoholic fatty liver disease (NAFLD) [5,6].

NAFLD describes a range of liver pathologies that have in common a lipid accumulation in hepatocytes in the absence of significant alcohol intake. NAFLD is a major and growing health problem [7]. While in recent years the prevalence of NAFLD in the general population has attained a level of 25%, it is present in the vast majority of overweight or obese patients and is considered the hepatic manifestation of the metabolic syndrome [8]. The disease pattern ranges from fully reversible blunt steatosis (NAFL) to a chronically progressive disease (non-alcoholic steatohepatitis, NASH) that is characterized by varying degrees of hepatocyte death, inflammation, and fibrosis and may ultimately result in liver cirrhosis, hepatocellular carcinoma, and terminal organ failure [9]. The transition from steatosis to the more severe forms of the disease occurs in roughly one third of affected patients, but it is still unclear what triggers this progression. However, there is evidence that dietary cholesterol might impact this transition. A number of independent animal experimental studies showed that enrichment of a high fat diet with 0.2 to 2% cholesterol resulted in a rapid progression from blunt steatosis to a NASH-like phenotype with ballooning, infiltration with inflammatory cells, and fibrosis [10,11,12,13,14]. Similarly, animal experiments [15] and clinical studies [16] using the cholesterol uptake inhibitor ezitimibe suggest that inhibition of cholesterol uptake from the gut might protect against NASH development. The mechanisms of how dietary cholesterol might trigger the transition to NASH have so far not been fully elucidated. The supposed mechanisms include activation of ER-stress response in hepatocytes, impairment of mitochondrial function resulting in oxidative stress, and the activation of resident or infiltrating macrophages by danger-associated molecular patterns (DAMPs) released from cholesterol-laden dying hepatocytes [17,18].

Our current study supports the view that dietary cholesterol can trigger NASH-development by causing mitochondrial dysfunction and oxidative stress and shows that these patho-mechanisms are severely aggravated by the presence of poly-unsaturated fatty acids (PUFA) in dietary fat.

## 2. Materials and Methods

All chemicals were of analytical or higher grade and obtained from local providers unless otherwise stated.

Animals and experimental design. Male C57BL/6JRj mice (own breeding) were housed in type II-cages at 20 ± 2 °C with a 12 h light/dark-cycle. Mice were randomly assigned to one of the following diet groups with free access to food and drinking water for 20 weeks: standard chow diet (V153 R/M-H; Ssniff, Soest, Germany) (STD), 0.75% cholesterol on a standard diet (CHO + STD), 0.75% cholesterol in a high fat diet containing ω6-PUFA-rich soybean oil (CHO + SOY; Altromin, Lage, Germany) or 0.75% cholesterol in a high fat diet containing mainly lard as fat source (CHO + LAR, D12451; Research Diets, New Brunswick, NJ, USA). Detailed diet composition is shown in Table 1. Mice had access to wooden gnawing sticks to avoid excessive teeth growth. Body weight was measured weekly. Mice were killed by cervical dislocation after isoflurane anesthesia. Serum and organs were snap-frozen in liquid nitrogen and stored at −70 °C for biochemical analysis, aliquots of the organs were fixed for histological examination. Animal experiments were performed according to the ARRIVE guidelines. Treatment of the animals followed the German animal protection laws and was performed with approval of the state animal welfare committee (LUGV Brandenburg, V3 2347).

In vivo experiments. Body fat content was measured at the beginning and at the end of the diet intervention by nuclear magnetic resonance spectroscopy (EchoMRI 2012 Body Composition Analyzer, Houston, TX, USA). The oral glucose tolerance test was performed in week 18 after an overnight fast by oral gavage of glucose (2 mg/kg body weight). Glucose and insulin levels were measured at the times indicated by a glucose sensor (Breeze2, Bayer; Berlin, Germany) or an insulin ELISA kit (Crystall Chem; Downers Grove, IL, USA).

Serum and tissue analysis. Serum parameters were quantified by an automated analyzer (Cobas Mira S, Hoffmann-La Roche, Basel, Switzerland) with the appropriate commercially available reagent kits. Liver triglycerides were determined by TRIGS-assay (Randox; Crumlin, UK). Total and free cholesterol in liver tissue was determined by a modified version of a protocol described previously [19]. Briefly, frozen tissues were homogenized by sonication using phosphate buffer (10 mM, pH 7.4) containing 1% polyoxyehylen-10-tridecylether. Homogenates were heated (5 min at 70 °C) to inactivate enzymes and then centrifuged for 10 min at 4 °C. Aliquots of supernatant were incubated in the presence or absence of 0.5 U/mL of cholesterol esterase to quantify the total and free cholesterol, respectively. The reaction buffer contained 100 mmol/L Tris (pH 7.7); 6 mmol/l phenol, 1 mmol/L 4-aminoantipyrine, 4 mmol/L 3,4-dichlorophenol, 10 mmol/L sodium cholate, 3 g/L fatty alcohol polyglycol ether, 50 mmol/L MgCl_2_, 0.2 U/mL cholesterol oxidase, and 0.4 U/mL peroxidase. The quinoneimine dye formed after 30 min is proportional to the quantity of cholesterol and was detected at 500 nm. A calibration curve was performed using a cholesterol solution 200 mg% (m/v) in fatty alcohol polyglycol ether (3 g/L). The esterified cholesterol was quantified by the difference between total and free cholesterol. Malondialdehyde was quantified by HPLC with fluorescence detection as described previously [11]. 

Histology. Formalin-fixed and paraffin-embedded liver sections (2–3 µm) were stained with Hematoxylin & Eosin or Sirius Red (both Sigma-Aldrich, Taufkirchen, Germany). Immunohistochemistry analyses were performed with anti-F4/80 antibody (AbD Serotec, Bio-Rad, Munich, Germany). Terminal deoxynucleotidyl transferase dUTP Nick End Labeling (TUNEL) assay was achieved with the Click-iT™ TUNEL Colorimetric IHC Detection Kit (Thermo Fisher Scientific, Berlin, Germany). Histological steatosis, inflammation and fibrosis were graded according to the NASH activity score (NAS) [20,21] by a liver pathologist (KJ) blinded to the diet. Quantification of histological staining of Sirius Red, F4/80, and TUNEL-positive cells was performed by using ImageJ software (version ImageJ 1.51j8, Wayne Rasband, National Institutes of Health, USA) in images of five randomly chosen fields of each liver. Details are described in the Methods section of the Supplementary Material. 

Real-time RT-PCR analysis. RNA isolation, reverse transcription, and qPCR were performed as previously described [22]. Results are expressed as relative gene expression normalized to expression levels of reference genes (Hprt, Eef2 and Srsf4) according to the formula: fold induction = 2 ^(a − b) gene of interest^/2 ^(a − b) reference genes^. Parameter “a” is the arithmetic mean of all Ct-values from samples of the STD group and parameter “b” is the Ct-value of every single sample. For calculations with more than one reference gene the geometric mean of the difference (a − b) of each reference gene was used.

Western blot and Oxyblot analysis. Western blot was performed as described previously [23] with anti-PGC-1α antibody and oxidative phosphorylation cocktail for Western blot (both abcam, Cambridge, UK), as well as Ponceau S-staining (Sigma-Aldrich, Taufkirchen, Germany) as a loading control. Oxyblot analysis was done as described [11] with anti-DNP antibody (Sigma-Aldrich, Taufkirchen, Germany). Visualization of immune complexes was performed by using a chemoluminescence reagent in the ChemiDoc™ Imaging System with ImageLab software (Bio-Rad, Munich, Germany). 

Statistical analysis. The statistical significance of differences was determined by one-way-ANOVA with Tukey’s post hoc test for multiple comparisons or Krukal-Wallis test for non-parametric samples as detailed in the legends to the figures using GraphPad Prism version 6 for Windows (GraphPad Software, La Jolla, California, CA, USA). Differences with a *p* ≤  0.05 were considered statistically significant.

## 3. Results

### 3.1. Diet-Induced Weight Gain, Insulin Resistance and NAFLD

**Figure 1:**
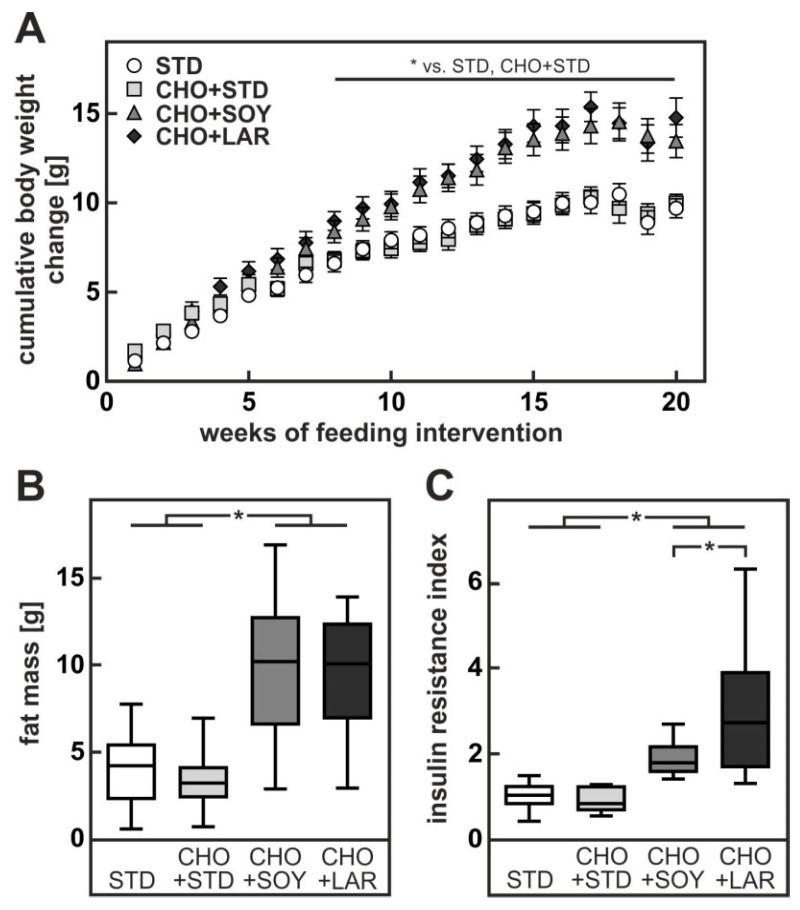
Increase in body weight, fat mass and insulin resistance in mice fed a CHO + SOY or CHO + LAR diet for 20 weeks. (**A**) Cumulative body weight change. (**B**) Fat mass in week 20. (**C**) Insulin resistance index was calculated by the sum of the products of insulin concentration × glucose concentration during the oral glucose tolerance test. Values are median (line), upper- and lower quartile (box) and extremes (whiskers) of 17–35 (**A**,**B**) or 8–10 (**C**) mice per group. Statistics: Multiple Student’s *t*-test for unpaired samples (**A**) or one-way-ANOVA with Tukey’s post hoc test for multiple comparisons (**B**,**C**). * *p* < 0.05.

Mice received either standard chow diet (STD), chow diet enriched with 0.75% cholesterol (CHO + STD), a soybean oil-based high fat diet with 0.75% cholesterol (CHO + SOY) or a lard-based high fat diet with 0.75% cholesterol (CHO + LAR) for 20 weeks, as described in Table 1. Animals on both high fat diets gained more weight than animals fed either chow diet or cholesterol-enriched chow diet (Figure 1A). The high fat diet-induced weight gain could be attributed to an increase in fat mass (Figure 1B) while the fat-free mass remained largely unaltered. Despite similar weight gain and increase in fat mass, animals fed the CHO + LAR diet were significantly more insulin resistant than animals receiving CHO + SOY diet (Figure 1C). As expected from the body weight data, CHO + STD-fed animals showed no signs of insulin resistance. 

**Figure 2:**
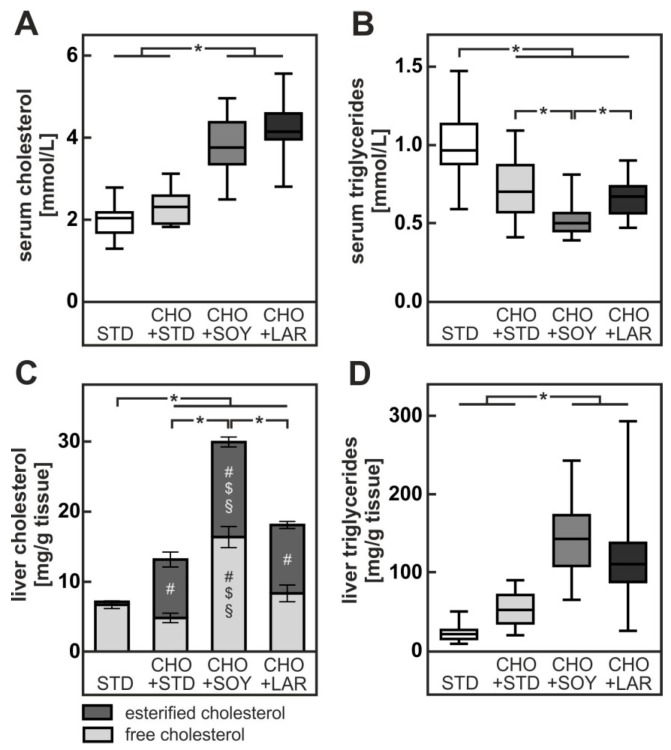
Diet-induced changes in serum and liver lipids after 20 weeks. (**A**) Cholesterol concentrations in serum. (**B**) Triglyceride concentrations in serum. (**C**) Levels of free and esterified cholesterol in liver. (**D**) Triglyceride levels in liver. Values are median (line), upper- and lower quartile (box) and extremes (whiskers) (**A**,**B**,**D**) or mean and sem (**C**) of 17–35 mice per group. Statistics: One-way-ANOVA with Tukey’s post hoc test for multiple comparisons. *: *p* < 0.05. Separate statistic for free and esterified cholesterol (**C**): #: vs. STD, $: vs. CHO + STD, §: vs. CHO+ LAD with *p* < 0.05.

Serum cholesterol levels increased only slightly (20%) in animals receiving the CHO + STD diet (Figure 2A). By contrast, serum cholesterol concentrations were doubled in comparison to the control in animals receiving either one of the high fat diets with cholesterol. Notably, no difference in serum cholesterol levels was observed between CHO + SOY- and CHO + LAR-fed animals (Figure 2A).

Unexpectedly, but in keeping with data of many independent studies in the literature [11,24,25], serum triglyceride levels were not elevated but instead were decreased in animals receiving either one of the cholesterol-enriched diets, irrespective of their fat content (Figure 2B). Total cholesterol was increased in livers of all animals receiving cholesterol-enriched diets. However, whereas CHO + STD and CHO + LAR-fed animals showed a similar approximately 2 to 3-fold increase in hepatic cholesterol content, animals receiving CHO + SOY diet exhibited a 6-fold increase in hepatic total cholesterol content (Figure 2C). Notably, free cholesterol was not significantly increased in CHO + STD-fed or CHO + LAR-fed animals in comparison to STD-fed animals, whereas free cholesterol content was doubled in CHO + SOY-fed mice (Figure 2C). In line with this, high amounts of cholesterol crystals could be detected only in livers of CHO + SOY-fed mice whereas only few or no cholesterol crystals were visible in livers of CHO + STD or CHO + LAR-fed mice (own observation).

Although weight gain was unaltered in animals receiving CHO + STD diet, these animals had a pronounced hepatic steatosis (see below, Figure 4). Hepatic triglyceride content increased more than twofold in comparison to chow-fed animals (Figure 2D). Hepatic triglyceride accumulation was more pronounced in animals receiving cholesterol-enriched high fat diets. Livers of CHO + SOY-fed and CHO + LAR-fed animals contained 7-fold or 5-fold more triglycerides than STD-fed animals, respectively (Figure 2D). The difference between the two high fat diets was, however, not significant. 

In summary, mice fed a CHO + SOY diet accumulated significantly higher amounts of free and esterified cholesterol in the liver compared to mice fed one of the other cholesterol-containing diets. Since the CHO + STD, CHO + SOY and CHO + LAD diets contained equal amounts of cholesterol, the combination of dietary cholesterol and ω6-PUFA-rich soybean oil may favor hepatic cholesterol accumulation.

**Figure 3:**
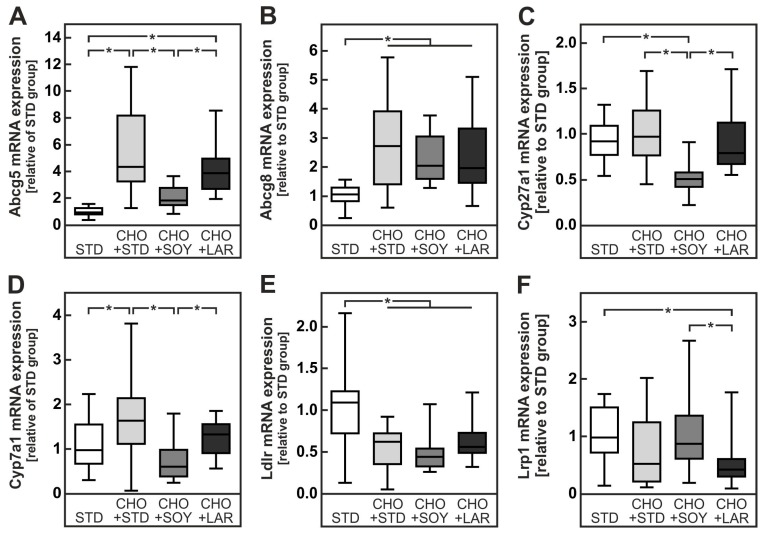
Markers of cholesterol metabolism in mice fed a cholesterol-containing diet for 20 weeks. Relative mRNA expression of the cholesterol transporters ATP-binding cassette sub-family G (Abcg) member 5 (**A**) and 8 (**B**), the cholesterol- metabolizing enzymes cytochrome P450 family 27 a1 (Cyp27a1, **C**) and family 7 a1 (Cyp7a1, **D**) as well as the transporters for the cholesterol intake LDL receptor (Ldlr, **E**) and LDL receptor related protein 1 (Lrp1, **F**) in mice liver. Values are median (line), upper- and lower quartile (box) and extremes (whiskers) of 17–35 mice per group. Statistics: One-way-ANOVA with Tukey’s post hoc test for multiple comparisons. *: *p* < 0.05.

The more pronounced increase in hepatic cholesterol content can either be the consequence of an enhanced uptake or a diminished excretion or conversion of cholesterol. In accordance with the latter hypothesis, the expression of the cholesterol export pump Abcg5 was induced more than fourfold in animals receiving either CHO + STD or CHO + LAR diet (Figure 3A). By contrast, the export pump was induced merely twofold, and hence significantly less, in animals receiving CHO + SOY diet than in either of the other two cholesterol containing diets (Figure 3A). Gene expression of Abcg8, the heterodimerization partner of Abcg5, was similar, yet it did not reach significance (Figure 3B). In comparison to the standard chow diet, Abca1, another cholesterol transporter mainly expressed in macrophages, was induced approximately 1.32-fold in livers of mice fed any of the cholesterol-containing diets. The increase was significant only in the CHO + STD diet group and no significant differences between the cholesterol-fed groups were observed.

In addition, the expression of Cyp27a1, a key enzyme for the conversion of cholesterol into bile acids, was significantly repressed in livers of CHO + SOY-fed mice (Figure 3C). Similarly, Cyp7a1 was repressed in CHO + SOY-fed animals, whereas it was unaffected or even induced in CHO + LAR diet and CHO + STD diet-fed animals, respectively (Figure 3D). Gene expression of the LDL receptor was repressed to a similar extent in livers of animals receiving any of the three cholesterol-containing diets (Figure 3E). By contrast, the expression of the LDL receptor related protein 1 (Lrp1) was slightly or significantly reduced in livers of animals receiving the CHO + STD or CHO + LAR diets, whereas expression was unaltered in livers of CHO + SOY-fed animals (Figure 3F).

In conclusion, the enhanced cholesterol accumulation in livers of CHO + SOY-fed mice can be explained by a decreased Abcg5-mediated cholesterol export, reduced Cyp27a1-dependent conversion of cholesterol into bile acids, as well as impaired repression of Lrp1-related cholesterol uptake into hepatocytes.

**Figure 4:**
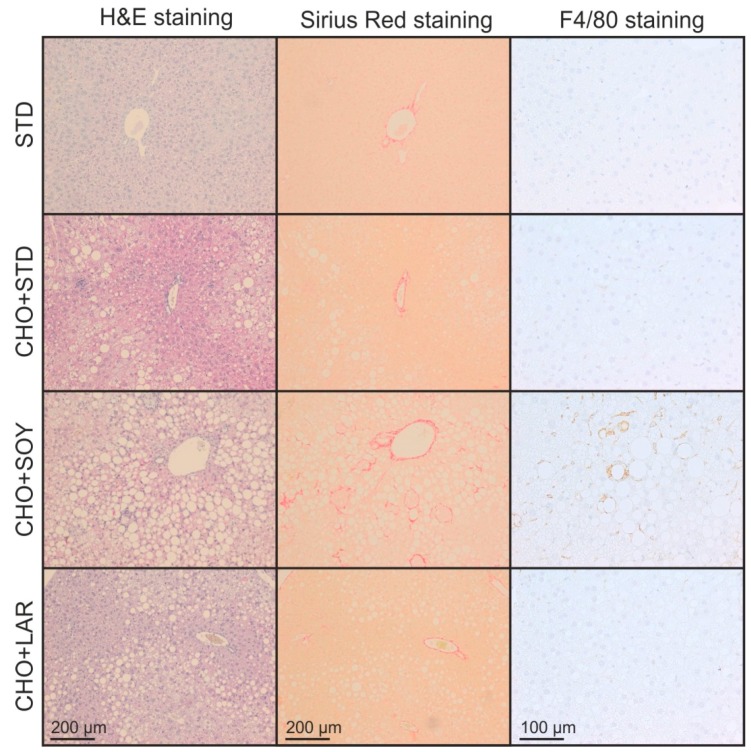
CHO + SOY diet induced steatohepatitis with steatosis, fibrosis, and macrophage infiltration. Mice received the diets for 20 weeks. Representative microphotographs of liver sections, magnification 10× or 20×.

Following, livers were examined histologically to determine the NASH activity score (NAS) (Table 2 and Figure 4). No signs of NAFLD were detected in livers of STD-fed control animals. By contrast, all animals receiving cholesterol-enriched diets had a positive NAS. However, while the average NAS for CHO + STD-fed and CHO + LAR-fed animals reached a maximum of 4 and hence indicated the presence of blunt steatosis, the average NAS of CHO + SOY-fed mice was above 7, clearly indicating the presence of active NASH (Table 2).

### 3.2. Diet-Induced Inflammation and Fibrosis

**Figure 5:**
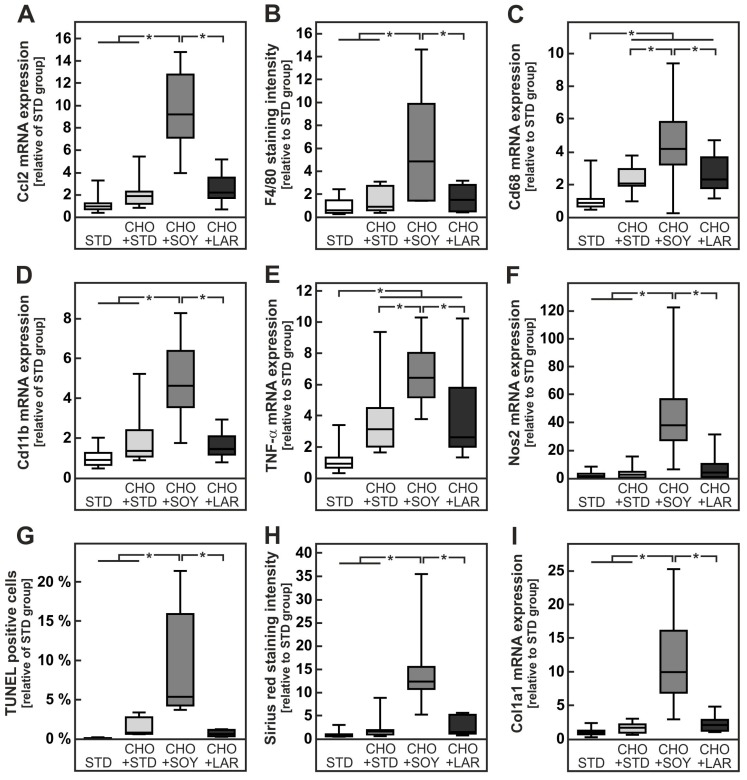
Enhanced macrophage infiltration, inflammation, apoptosis and fibrosis in mice fed a CHO + SOY diet. Mice received the diets for 20 weeks. (**A**) Relative mRNA expression of the chemokine Ccl2 (alternative name Mcp-1) in mice liver. (**B**) Quantification of F4/80-stained microphotographs of the liver. (**C**,**D**,**E**,**F**) Relative mRNA expression of the macrophage markers Cd68 (**C**) and Cd11b (**D**, gene name *Itgam*), the cytokine tumor necrosis factor α (TNF- α, **E**) and the enzyme inducible nitric oxide synthase 2 (**F**, alternative name iNos) in mice liver. (**G**) Quantification of hepatocyte apoptosis by TUNEL assay. (**H**) Quantification of Sirius Red-stained microphotographs of the liver calculated by dense intensity of Sirius Red relative to the amount of cytosolic background per field in 5 randomly chosen microphotographs per liver section. (**I**) Relative mRNA expression of the fibrosis marker collagen 1a1 (Col1a1) in mice liver. Values are median (line), upper- and lower quartile (box) and extremes (whiskers) of 17–35 (**A**,**C**,**D**,**E**,**F**,**I**), 4–7 (**B**,**G**) or 13–16 (**H**) mice per group. Statistics: One-way-ANOVA with Tukey’s post hoc test for multiple comparisons. *: *p* < 0.05.

All cholesterol-containing diets apparently triggered an inflammatory response in the liver. However, in accordance with the higher NAS, animals receiving the CHO + SOY diet showed more pronounced signs of inflammation (Figure 4 right panel, quantification in Figure 5B). The expression of the chemotactic cytokine Ccl2 (Mcp-1) was increased about two-fold in animals receiving CHO + STD or CHO + LAR diets, whereas an almost 10-fold increase was observed in CHO + SOY diet-fed animals (Figure 5A). Consequently, the expression of the macrophage markers F4/80, Cd68, and Cd11b was increased by all cholesterol-containing diets, but was significantly higher in CHO + SOY-fed animals than in animals receiving any of the other diets (Figure 5B–D). Similarly, the induction of the pro-inflammatory cytokine TNF-α was two-fold higher in livers of CHO + SOY-fed mice than in animals that received the CHO + STD or CHO + LAR diet (Figure 5E). Inducible nitric oxide synthase (Nos2, iNos), a key enzyme in inflammation-dependent NO production was only induced in livers of CHO + SOY-fed animals (Figure 5F). Furthermore, a significantly higher amount of TUNEL-positive hepatocytes were detected in livers of CHO + SOY-fed mice compared to mice fed any of the other diets, showing increased hepatic apoptosis (Figure 5G). 

In order to assess fibrosis, liver slices were stained with Sirius Red (Figure 4). A significant increase in fibrosis was only observed in animals receiving CHO + SOY diet, whereas all other animals only showed minor age-appropriate positive staining for Sirius Red (Figure 5H). In accordance with these histological data, collagen 1a1 expression was slightly induced in animals fed CHO + STD or CHO + LAR diets, whereas a more than 10-fold induction was observed in livers of CHO + SOY-fed mice (Figure 5I).

These results show that only mice fed a CHO + SOY diet developed clear signs of hepatic inflammation with macrophage infiltration and increased expression of pro-inflammatory cytokines as well as hepatocyte apoptosis and liver fibrosis. 

### 3.3. Diet-Induced Mitochondrial Damage and Oxidative Stress

**Figure 6:**
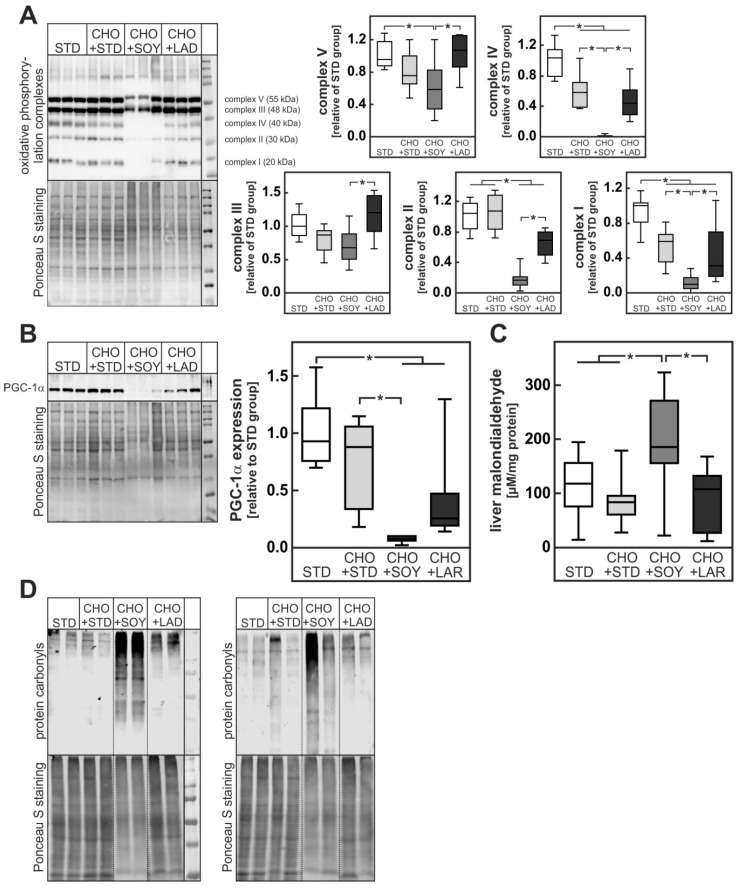
Increased mitochondrial damage and oxidative stress in mice fed a CHO + SOY diet for 20 weeks. (**A**,**B**) Hepatic protein expression of the oxidative phosphorylation complexes (**A**) and PGC-1α (**B**). Dense intensity was normalized to Ponceau S staining, which was verified on the same Western blot membrane as a loading control and calculated relative to the STD group in each gel. A representative blot is shown. All original blots are provided in the Supplementary Material, Figures S1 and S2. (**C**) Concentration of malondialdehyde in liver as a marker of lipid peroxidation. (**D**) Determination of protein carbonyls in liver homogenates verified by oxyblot with Ponceau staining as a loading control. Blots were cut at the dotted lines. Original blots are shown in the Supplementary Material, Figure S3. Values are median (line), upper- and lower quartile (box) and extremes (whiskers) of 8–10 (**A**,**B**) or 9–28 (**C**) mice per group. Statistics: One-way-ANOVA with Tukey’s post hoc test for multiple comparisons. *: *p* < 0.05.

The cholesterol-dependent induction of liver damage has previously been attributed to mitochondrial damage resulting from cholesterol accumulation in mitochondrial membranes. However, judging from the expression of complex I, II, and IV of the respiratory chain, dietary cholesterol alone apparently did cause low but no severe mitochondrial damage in our model (Figure 6A). A mild reduction of complex I, II, and IV content was also observed in livers from animals receiving CHO + LAR diet. In stark contrast, complex I, II, and IV proteins were dramatically reduced in livers of CHO + SOY-fed animals, indicating severe mitochondrial damage in only this group (Figure 6A). In keeping with these data, the amount of PGC-1α protein was strongly reduced in livers of animals receiving the CHO + SOY diet (Figure 6B). 

Impairment of mitochondrial respiration causes severe oxidative stress. Accordingly, levels of malondialdehyde, a reaction product of lipid peroxidation of unsaturated fatty acids, and protein carbonyls were only increased in livers of CHO + SOY-fed mice (Figure 6C,D).

Thus, cholesterol-induced mitochondrial damage and oxidative stress was clearly enhanced by soybean oil-derived PUFA probably due to augmented lipid peroxidation. 

### 3.4. Oxidative Stress Preceding Inflammation

**Figure 7:**
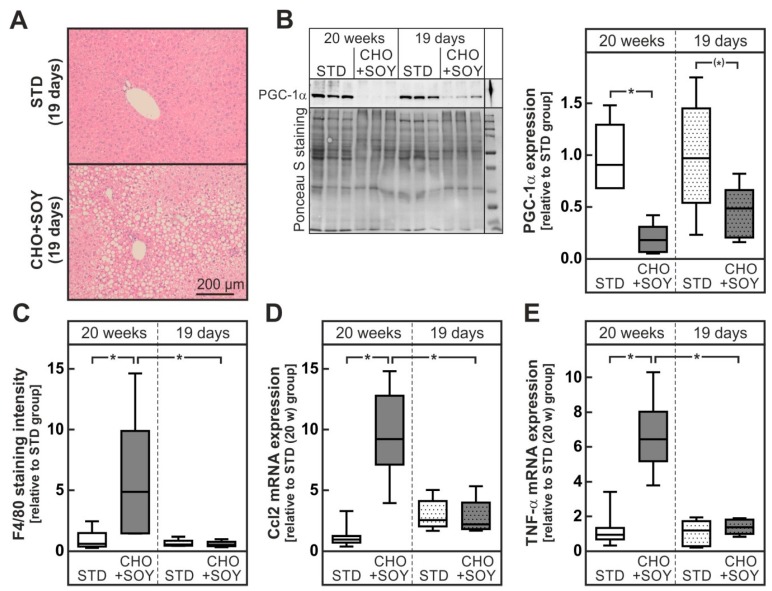
Enhanced mitochondrial damage but no signs of inflammation in mice fed a CHO + SOY diet for 19 days. Mice received the STD CHO + SOY diet for 20 weeks or 19 days. (**A**) Representative H&E-stained microphotographs of the liver. (**B**) Hepatic protein expression of PGC-1α after 20 week and 19 days feeding intervention. Dense intensity was normalized to Ponceau S staining, which was verified on the same Western blot membrane as a loading control and calculated relative to the STD group in each gel. A representative blot is shown. All original blots are provided in the Supplementary Material, Figure S4. (**C**) Quantification of F4/80-stained microphotographs of the liver calculated by dense intensity of F4/80 relative to the amount of cytosolic background per field in 5 randomly chosen microphotographs per liver section. (**D**,**E**) Relative mRNA expression of Ccl2 (**D**, alternative name Mcp-1) and tumor necrosis factor α (TNF-α, E) in mice liver. Values are median (line), upper- and lower quartile (box) and extremes (whiskers) of 6 (**B**,**C**) or 6–7 (**D**,**E**) mice per group. Statistics: One-way-ANOVA with Tukey’s post hoc test for multiple comparisons. *: *p* < 0.05.

In order to elucidate whether oxidative stress in livers of CHO + SOY-fed animals was the cause or consequence of the inflammation observed in the livers of these animals, the CHO + SOY diet was fed for a shorter period, i.e., 19 days. While liver steatosis, a reduction of PGC-1α, a reduction of complexes of the respiratory chain as well as signs of oxidative stress were already present after 19 days of feeding, no increase in inflammatory markers was detectable, indicating that mitochondrial damage and oxidative stress precede the development of inflammation (Figure 7, Figure S4 in Supplementary Material).

## 4. Discussion

In our current study we showed that dietary cholesterol induced hepatic steatosis and NAFLD independent of the accompanying lipid content of the diet. However, histological signs of progression to NASH, hepatocyte apoptosis, infiltration with inflammatory cells, and fibrosis only developed when dietary cholesterol was combined with a soybean oil-based high fat diet rich in ω6-PUFA. Our results indicate the contribution of two potentially cooperating mechanisms to this transition: (1) soybean oil-derived PUFA increased hepatic cholesterol accumulation, most likely by repression of pathways that are responsible for the elimination of cholesterol via the bile. (2) soybean oil-derived PUFA augmented cholesterol-induced mitochondrial damage and oxidative stress presumably via lipid peroxidation.

### 4.1. PUFA-Dependent Increase in Cholesterol Accumulation

An increase in hepatic cholesterol accumulation by a soybean oil-based high fat diet has been observed previously in a study that compared the tissue distribution of cholesterol in rats fed a soybean oil-based or tallow-based high fat diet [26]. Yet, the underlying mechanisms were not analyzed in this early study. Hepatic cholesterol accumulation in the presence of dietary PUFA could result either from increased hepatic uptake or decreased removal. 

Since dietary cholesterol is absorbed via the chylomicron pathway, an increase in hepatic cholesterol uptake should most likely be mediated by an augmented hepatic clearance of remnant particles or absorption of HDL cholesterol. Both processes involve the LDL receptor, the LDL-receptor-related protein 1, and the scavenger receptor class B type1. While the LDL receptor, as expected, was downregulated in animals fed any of the cholesterol-containing diets, gene expression of the LDL receptor related protein 1 (Lrp1) was reduced only in animals receiving CHO + STD or CHO + LAR diets, but not in the CHO + SOY-fed animals (Figure 3E,F). These differences were however rather small and unlikely to account for the two-fold increase in hepatic cholesterol observed in CHO + SOY-fed animals (Figure 2C). In the literature, a PUFA-induced increase in SR-B1 expression was described in a genetically obese rat strain [27] that was supposed to result in a more efficient hepatic uptake of cholesterol from HDL and hence might contribute to hepatic cholesterol accumulation and at the same time contribute to the anti-atherogenic effect of dietary PUFA. 

The current data indicate that a dietary PUFA-dependent inhibition of cholesterol removal from the liver either as free cholesterol or after conversion into bile acids is the more likely explanation for the pronounced cholesterol accumulation in livers of CHO + SOY-fed animals. Cholesterol can be excreted directly into the bile by the ABCG5/ABCG8 export pump [28]. The expression of Abcg5 was induced four- to sixfold in CHO + LAR- or CHO + STD-fed animals, respectively. By contrast a significantly lower twofold induction was observed in livers of CHO + SOY-fed animals (Figure 3A,B). Similarly, the expression of the genes for enzymes involved in bile acid formation was repressed by CHO + SOY diet feeding. The CHO + SOY diet is rich in ω6-PUFA whereas the content in ω3-fatty acids is relatively low. In contrast to ω6-PUFA, ω3-PUFA appeared to increase the expression both of cholesterol export pumps and key enzymes of bile acid synthesis [29]. Interestingly, whereas ω6-fatty acids favor accumulation of cholesterol in the liver, long chain ω3-poly-unsaturated fatty acids seem to counteract this effect. Thus, hamsters fed a diet that contained 38% linoleic acid (18:2(ω6)) accumulated almost twice as much cholesterol ester in the liver as animals fed a similar diet in which half of the linoleic acid was replaced by long chain ω3-fatty acids, mainly eicosapentaenoic acid (20:5(ω3)) and docosahexaenoic acid (22:6(ω3)) [30]. Similarly, hepatic cholesterol content was reduced by long chain ω3-PUFA supplementation in mice fed a high fat high cholesterol diet rich in ω6-fatty acids [31]. This might in part explain why supplementation with ω3-fatty acids has repeatedly been reported to protect from NAFLD or NASH development [32,33] while depletion of ω3-PUFA increased hepatic steatosis [34].

Also highly unlikely, it cannot be entirely excluded that apart from the pronounced differences in the fatty acid composition minor differences in the protein composition between the two high fat diets also contributed to the different phenotypes observed in particular to hepatic lipid accumulation, since diets extremely rich in protein appear to protect from hepatic steatosis [35].

### 4.2. PUFA-Dependent Enhancement of Oxidative Stress

Dietary cholesterol initially accumulates in the hepatocyte. This results in an increase in cholesterol content in the membranes of different cellular compartments and the subsequent impairment of their function. Thus, it has been shown that an increment in the cholesterol content of the ER membrane may result in the inhibition of the ER calcium pump, a drop in ER calcium concentration, impaired protein folding, and ER stress ([18] and references therein). It has, however, been questioned, whether this mechanism is relevant for NASH development [36]. Similarly, accumulation of cholesterol in the outer phospholipid monolayer of lipid droplets has been assumed to impair lipid turnover and may result in the formation of cholesterol crystals in lipid droplets [12]. Most importantly, however, excessive incorporation of cholesterol in mitochondrial membranes has been shown to impair the function of the α-ketoglutarate carrier that is responsible for the import of reduced glutathione from the cytosol into the mitochondrion [37]. As a consequence, the quenching of reactive oxygen species formed in the respiratory chain is impaired and oxidative stress ensues [38]. The oxidative stress further impairs mitochondrial function and may sensitize the hepatocyte to other death-inducing signals. Apparently, this mechanism was further aggravated by the presence of PUFA in the CHO + SOY diet, since only the combination of cholesterol with soybean oil-based high fat diet resulted in a reduction of mitochondrial respiratory chain proteins and PGC-1α, the master regulator of mitochondrial biogenesis (Figure 6A,B), as well as profound oxidative stress leading to the formation of large amounts of protein carbonyls (Figure 6D). Apart from enhancing cholesterol accumulation in the hepatocyte (see above), PUFA might amplify cholesterol-dependent oxidative stress by lipid peroxidation chain reactions resulting, among others, in the formation of malondialdehyde (Figure 6C). Lipid peroxides have been shown to decrease the content of mitochondrial respiratory chain proteins [39] and cause mitochondrial dysfunction. A reduction in the content of mitochondrial respiratory chain proteins in the liver of NASH patients has been described [25]. Lipid peroxidation also favors the non-enzymatic formation of oxysterols. While oxysterols at low concentrations activate liver X receptor (LXR) and may initiate pathways that protect against consequences of a cholesterol overload, high oxysterol concentrations induce apoptosis by triggering the mitochondrial apoptotic pathway [40] in hepatoma cells or primary rat hepatocytes, in particular if cells were exposed to a combination of oxysterols and fatty acids. In addition, oxysterols at high concentrations appear to contribute to cell death by antagonizing Akt-dependent survival pathways [41]. Oxysterols are elevated in NAFLD patients [42] and may be causative in NASH development [43].

The oxidative stress-induced death of the cholesterol laden hepatocytes might then trigger the subsequent inflammatory response and the initiation of the development of fibrosis. Notably, signs of mitochondrial damage and oxidative stress preceded the development of inflammation (Figure 7). Hepatocyte detritus may be taken up by Kupffer cells and infiltrating macrophages that have been shown to form crown-like structures around dying hepatocytes [44]. Since cholesterol cannot be removed by macrophages, this results in a self-perpetuating chronic inflammation that triggers scar formation and fibrosis (Figure 4). Activation of macrophages and stellate cells by cholesterol or cholesterol crystals [11,12,45] and oxysterols [46] has been shown to promote this process. 

### 4.3. Possible Clinical Impact

There is evidence that dietary cholesterol may also favor NASH development in humans [47,48,49] and the interruption of intestinal cholesterol absorption by ezetimibe has been shown to be beneficial in NAFLD patients in a meta-analysis of several clinical studies [16]. Notably, in one study ezetimibe reduced the NASH activity score without affecting steatosis [50]. A possible interaction between dietary cholesterol and fatty acid composition of the diet apparently was not systematically analyzed in human studies. However, meta-analysis of several clinical studies showed that supplementation with long chain ω3-PUFA caused a more or less pronounced reduction in the plasma levels of alanine aminotransferase (ALAT), aspartate aminotransferase (ASAT), and γ-glutamyltransferase (GGT), indicating a reduction of liver damage. However, the actual NASH activity score was not determined [51]. 

## 5. Conclusions

A direct translation of the results of the current study to a dietary recommendation for humans is beyond any doubt inappropriate. However, the data suggest that the recommendation to replace saturated fat by fat from sources rich in ω6-PUFA without a simultaneous reduction of cholesterol intake may be sensible from the point of view of protection against cardiovascular diseases and possibly other consequences of the metabolic syndrome, however, may not be advisable from the point of view of NASH development. 

## Figures and Tables

**Table 1:** Diet composition. Mice diets used in the feeding experiment. Standard chow diet (STD), 0.75% cholesterol in a Standard diet (CHO + CHO), 0.75% cholesterol in a high fat diet containing ω6-PUFA-rich soybean oil (CHO + SOY), 0.75% cholesterol in a high fat diet containing mainly lard as fat source (CHO + LAR).

	STD	CHO + STD	CHO + SOY	CHO + LAR
Metabolizing energy (kcal/g)	3.06	3.06	4.64	4.73
Energy from carbohydrates (%)	65	65	35	35
Energy from protein (%)	25	25	16	20
Energy from fat (%)	10	10	49	45
Cholesterol (%)	0.00	0.75	0.75	0.75
Fatty acid composition				
Saturated fatty acids (g/100g)	0.55	0.55	4.00	7.26
Mono-unsaturated fatty acids (g/100g)	0.64	0.64	5.75	8.69
Poly-unsaturated fatty acids (g/100g)	2.01	2.01	14.50	6.36

**Table 2:** NASH activity score grading steatosis, ballooning (hepatocyte hypertrophy), inflammation, and fibrosis. Values are mean ± SEM of 17–35 mice per group. Statistics: Kruskal-Wallis test with Dunn’s post hoc test for multiple comparisons. #: vs. STD, $: vs. CHO + STD, §: vs. CHO + LAD with *p* < 0.05.

Scoring Parameter	STD	CHO + STD	CHO + SOY	CHO + LAR
Steatosis	0.00 ± 0.00	1.53 ± 0.37 (#)	3.77 ± 0.08 (#,$,§)	2.41 ± 0.33 (#)
Hepatocyte hypertrophy	0.00 ± 0.00	0.35 ± 0.15	1.73 ± 0.10 (#,$,§)	0.76 ± 0.18 (#)
Inflammation	0.10 ± 0.05	0.53 ± 0.15	1.50 ± 0.13 (#,$,§)	0.41 ± 0.12
Fibrosis	0.26 ± 0.08	0.59 ± 0.12	0.80 ± 0.07 (#)	0.47 ± 0.12
NASH activity score (NAS)	0.36 ± 0.10	3.00 ± 0.59 (#)	7.80 ± 0.20 (#,$,§)	4.06 ± 0.49 (#)

## Supplementary Materials

The following are available online at http://www.mdpi.com/2072-6643/10/9/1326/s1, Supplementary Methods: Quantification of immunohistochemistry analysis; Supplementary Figure S1: Expression of oxidative phosphorylation complexes in liver homogenates; Supplementary Figure S2: Expression of PGC-1α in liver homogenates; Supplementary Figure S3: Detection of protein carbonyls by oxyblot analysis in liver homogenates; Supplementary Figure S4: Increased mitochondrial damage and oxidative stress in mice fed a CHO + SOY diet for 19 days.
