# Supplementary material for: Soybean Oil-Derived Poly-Unsaturated Fatty Acids Enhance Liver Damage in NAFLD Induced by Dietary Cholesterol
Source: Nutrients. 2018 Sep 18;10(9):1326. doi: 10.3390/nu10091326 (PMC6164134; doi:10.3390/nu10091326)
Supplement: Supplementary file 1 [file nutrients-10-01326-s001.pdf]

Soybean oil-derived poly-unsaturated fatty acids enhance liver damage in NAFLD induced by dietary cholesterol

Janin Henkel, Eugenia Alfine, Juliana Saín, Korinna Jöhrens, Daniela Weber, José P. Castro, Jeanette König, Christin Stuhlmann, Madita Vahrenbrink, Wenke Jonas, André Kleinridders and Gerhard P. Püschel

SUPPLEMENTARY MATERIAL

Supplementary Methods

Quantification of immunohistochemistry analysis.

F4/80 immunohistochemistry analysis: ImageJ software was used and a macro was designed for automatic analysis of the dense intensity of immunohistochemistry staining of F4/80 using the 'histogram' tool. F4/80-positive areas were defined as sum of histogram data in the range of 120-210. Data were calculated relative to cytosolic, non-steatotic areas per field that were defined as mean dense intensity histogram data in the range of 220-240. Relative F4/80 dense intensity was calculated by dense intensity of F4/80 relative to the amount of cytosolic background per field in 5 randomly chosen microphotographs per liver section (Figure 5B and Figure 7C).

TUNEL-assay: A macro was designed for automatic analysis of the number of hepatocyte nuclei per field using ImageJ software. The following parameters were defined: 'medianRadius = 2' as well as 'size (pixel^2) = 1000' and 'circularity = 0.25-1.00' for the command 'analyze particles'. Threshold was adjusted to 'threshold = 1-185' for counting all hepatocyte nuclei and 'threshold = 1-160' for counting TUNEL-positive stained hepatocyte nuclei. For Quantification the number of TUNEL-positive cells relative to the number of hepatocyte nuclei per field was calculated in 5 randomly chosen microphotographs per liver section. Results are expressed by ratio of TUNEL-positive stained hepatocyte nuclei relative to all hepatocyte nuclei in % (Figure 5G).

Supplementary Figures

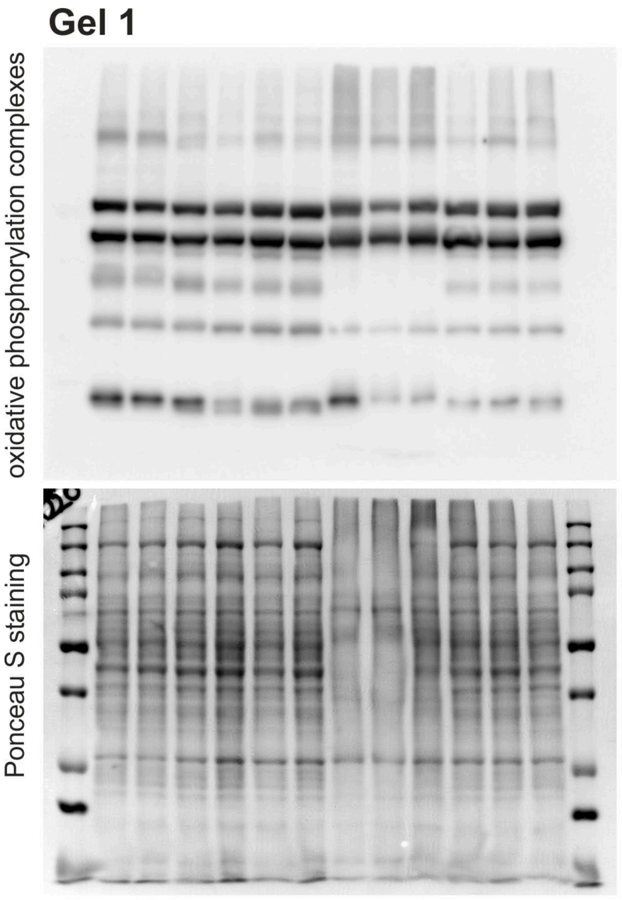

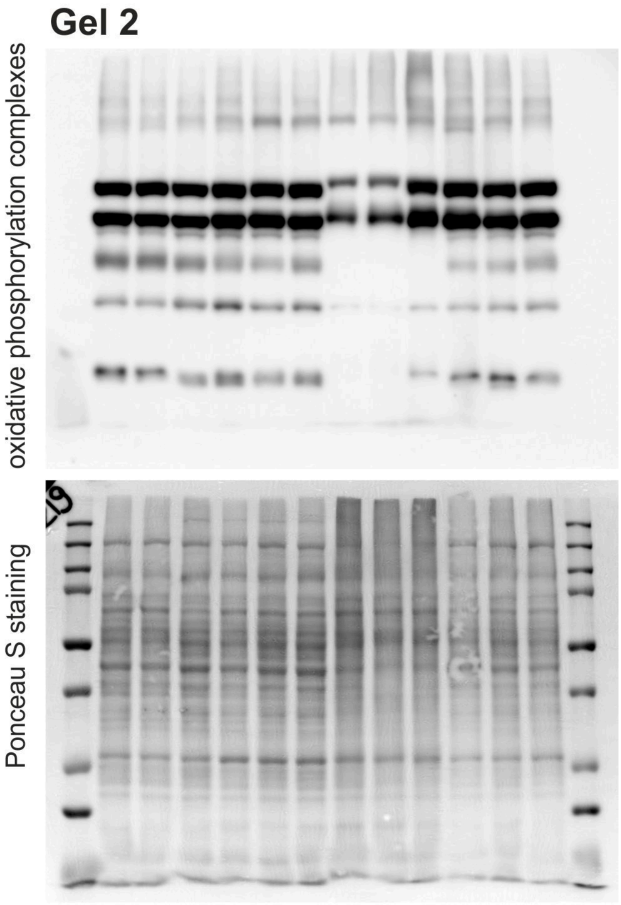

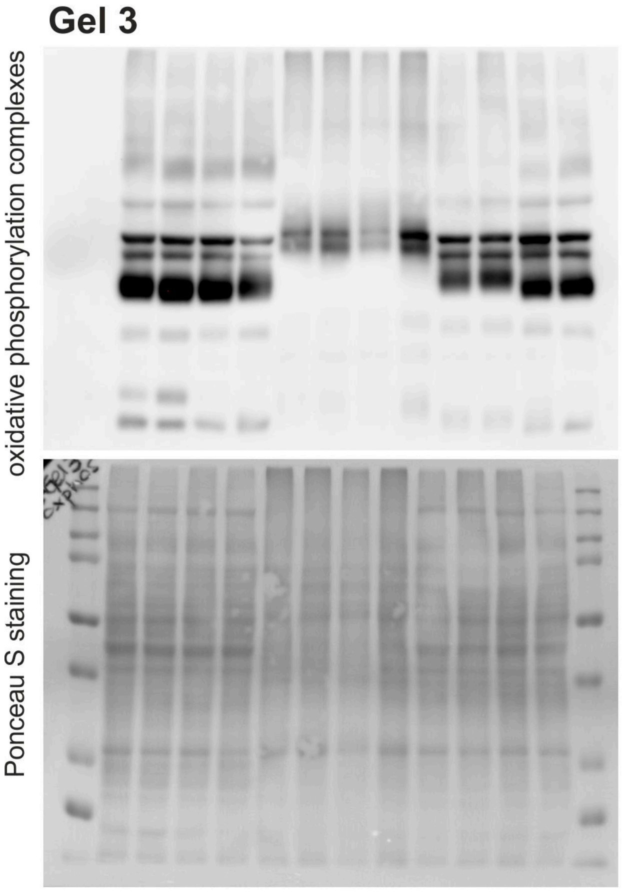

SAMPLE ORDER

Gel 1	3 x STD	3 x CHO +STD	3 x CHO +SOY	3 x CHO +LAR
Gel 2	3 x STD	3 x CHO +STD	3 x CHO +SOY	3 x CHO +LAR
Gel 3	2 x STD	2 x CHO +STD	4 x CHO +SOY	4 x CHO +LAR

MOLECULAR WEIGHT MARKER

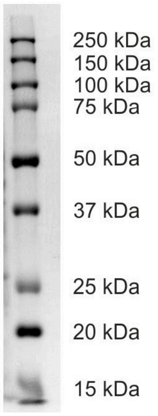

Supplementary Figure S1: Expression of oxidative phosphorylation complexes in liver homogenates.

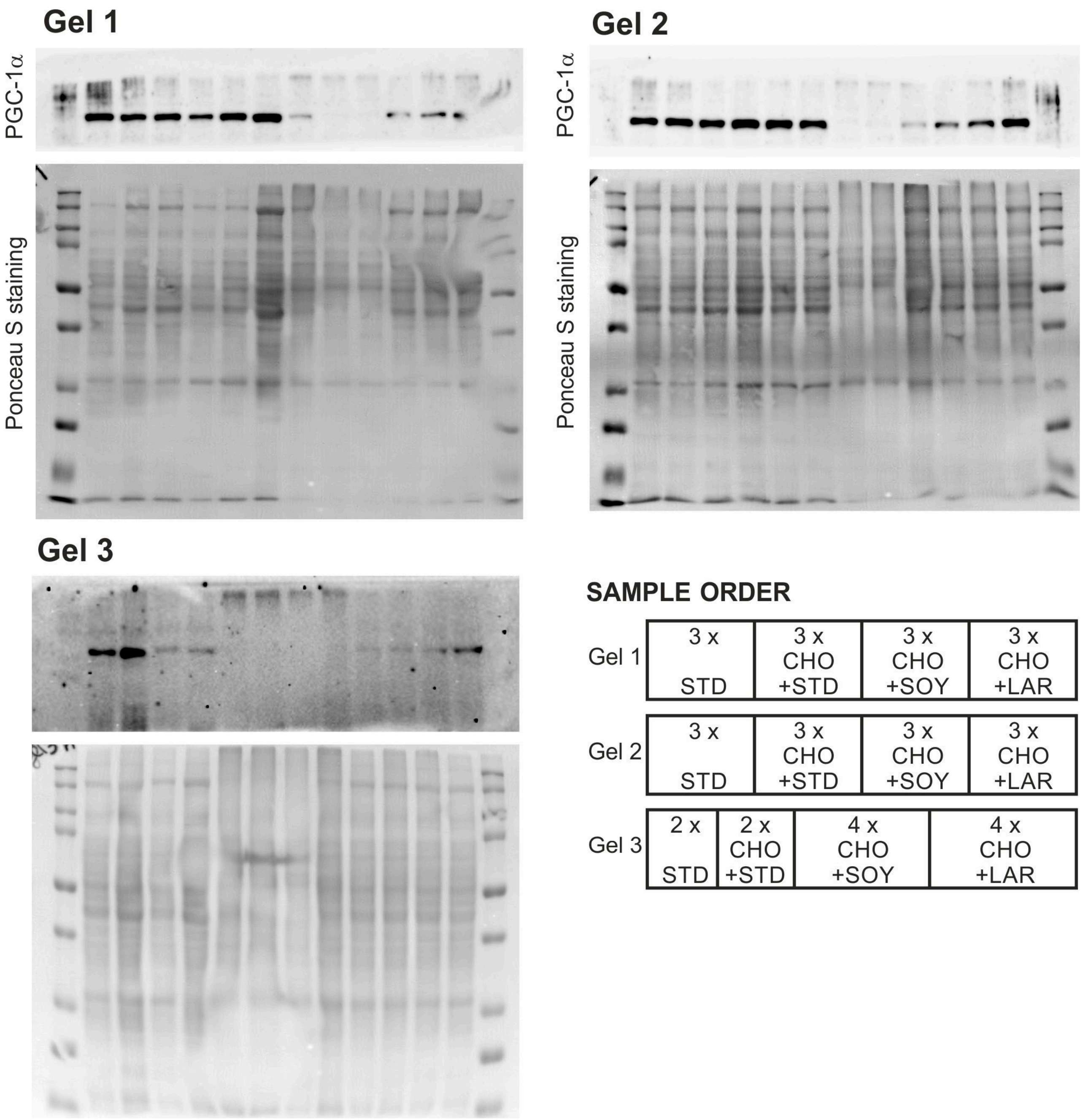

Supplementary Figure S2: Expression of PGC-1α in liver homogenates. For molecular weight marker details see Supplementary Figure S1.

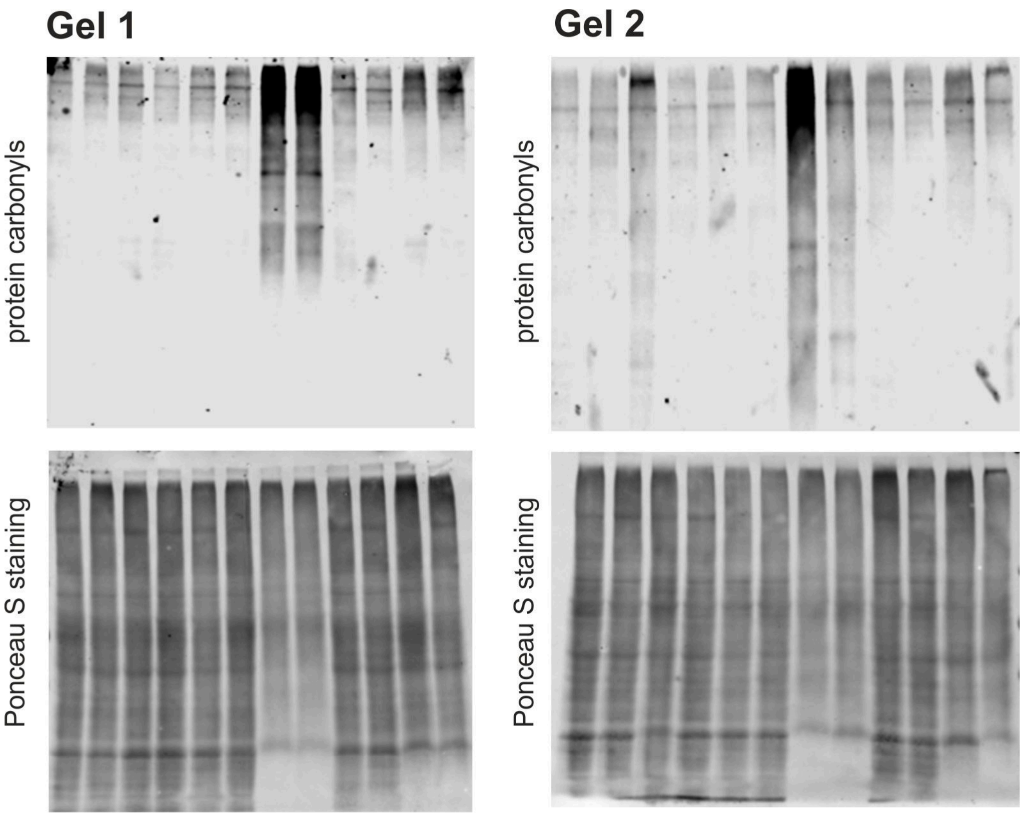

SAMPLE ORDER

Gel 1	2 x STD	2 x CHO +STD	2 x #	3 x CHO +SOY	2 x #	2 x CHO +LAR
Gel 1	2 x STD	2 x CHO +STD	2 x #	3 x CHO +SOY	2 x #	2 x CHO +LAR

#: not relevant samples

Supplementary Figure S3: Detection of protein carbonyls by oxyblot analysis in liver homogenates.

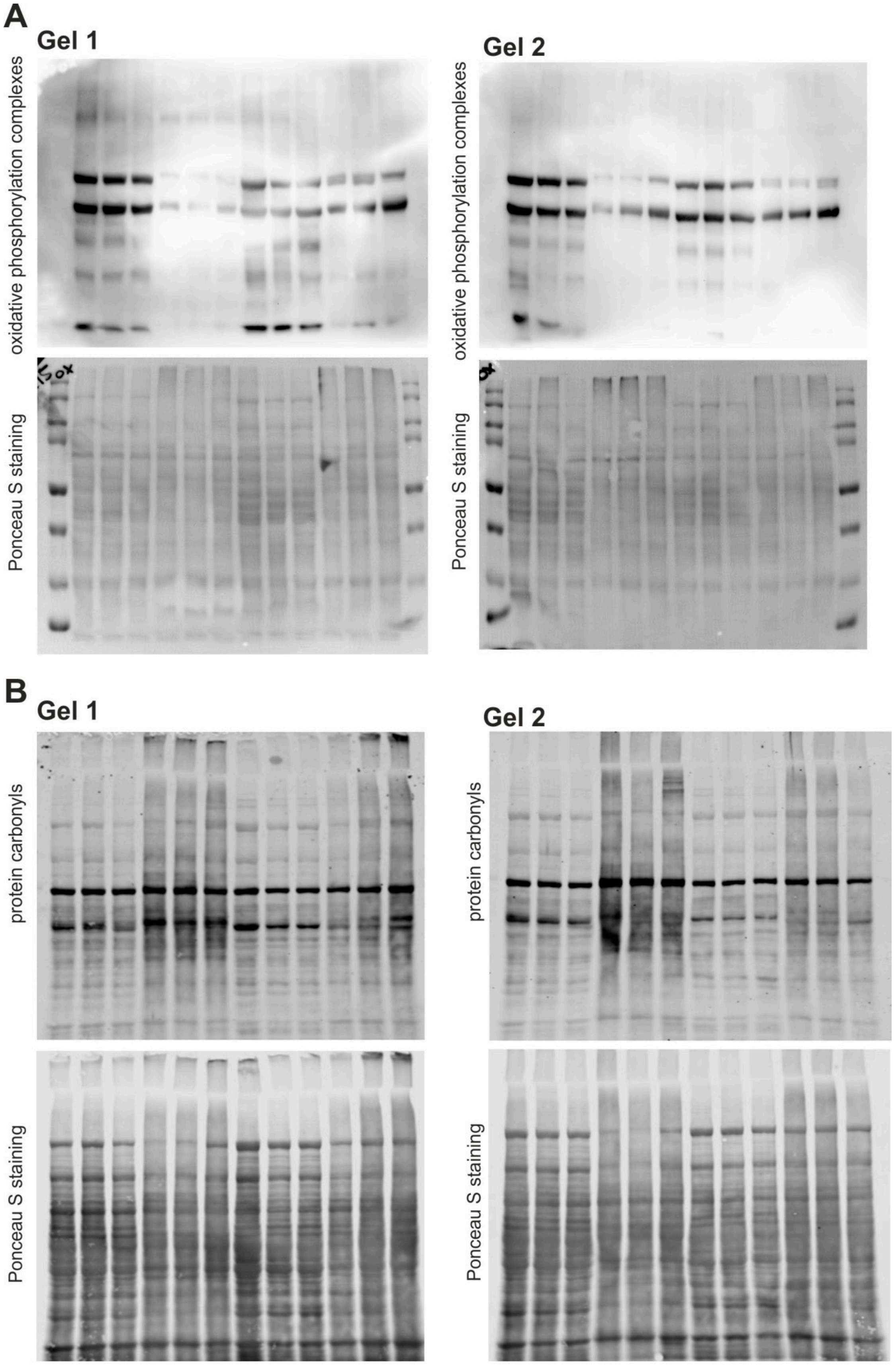

C

SAMPLE ORDER

Gel 1	3 x<br><br>STD<br>20 w	3 x<br>CHO<br>+SOY<br>20 w	3 x<br><br>STD<br>19 d	3 x<br>CHO<br>+SOY<br>19 d

SAMPLE ORDER

Gel 2	3 x<br><br>STD<br>20 w	3 x<br>CHO<br>+SOY<br>20 w	3 x<br><br>STD<br>19 d	3 x<br>CHO<br>+SOY<br>19 d

Supplementary Figure S4: Increased mitochondrial damage and oxidative stress in mice fed a CHO+SOY diet for 19 days. Hepatic protein expression of the oxidative phosphorylation complexes (A) and determination of protein carbonyls by oxyblot (B). For molecular weight marker details see Supplementary Figure S1.
